# The pain threshold of high-threshold mechanosensitive receptors subsequent to maximal eccentric exercise is a potential marker in the prediction of DOMS associated impairment

**DOI:** 10.1371/journal.pone.0185463

**Published:** 2017-10-06

**Authors:** Johannes Fleckenstein, Perikles Simon, Matthias König, Lutz Vogt, Winfried Banzer

**Affiliations:** 1 Department of Sports Medicine, Institute of Sports Sciences, Goethe-University Frankfurt, Frankfurt am Main, Germany; 2 Department of Sports Medicine, Johannes Gutenberg-University Mainz, Mainz, Germany; 3 Sport and Exercise Science Research Centre, School of Applied Sciences, London South Bank University, London, United Kingdom; 4 Institute of Movement and Sport Gerontology, German Sport University Cologne, Am Sportpark Müngersdorf 6, Cologne, Germany; INSEP, FRANCE

## Abstract

**Background:**

Delayed-onset muscle soreness (DOMS) refers to dull pain and discomfort in people after participating in exercise, sport or recreational physical activities. The aim of this study was to detect underlying mechanical thresholds in an experimental model of DOMS.

**Methods:**

Randomised study to detect mechanical pain thresholds in a randomised order following experimentally induced DOMS of the non-dominant arm in healthy participants. Main outcome was the detection of the pressure pain threshold (PPT), secondary thresholds included mechanical detection (MDT) and pain thresholds (MPT), pain intensity, pain perceptions and the maximum isometric voluntary force (MIVF).

**Results:**

Twenty volunteers (9 female and 11 male, age 25.2 ± 3.2 years, weight 70.5 ± 10.8 kg, height 177.4 ± 9.4 cm) participated in the study. DOMS reduced the PPT (at baseline 5.9 ± 0.4 kg/cm^2^) by a maximum of 1.5 ± 1.4 kg/cm^2^ (-24%) at 48 hours (p < 0.001). This correlated with the decrease in MIVF (r = -0.48, p = 0.033). Whereas subjective pain was an indicator of the early 48 hours, the PPT was still present after 72 hours (r = 0.48, p = 0.036). Other mechanical thresholds altered significantly due to DOMS, but did show no clinically or physiologically remarkable changes.

**Conclusions:**

Functional impairment following DOMS seems related to the increased excitability of high-threshold mechanosensitive nociceptors. The PPT was the most valid mechanical threshold to quantify the extent of dysfunction. Thus PPT rather than pain intensity should be considered a possible marker indicating the athletes’ potential risk of injury.

## Introduction

Delayed Onset Muscle Soreness (DOMS) has been subject to many studies investigating mechanism or treatments in exercise-related muscle pain, [1] and is a common state in elite and recreational athletes [2]. Mechanically-induced microtraumata, i.e. grade 1 muscle strain injuries, result in the perception of muscle soreness associated with pain and weakness [3]. The symptomology should be restricted to dull pain and discomfort, manifesting itself usually 6 to 12 hours and peaking 48 to 72 hours post-exercise [4, 5]. Often, people go to bed with mild discomfort and wake up the next morning with severe pain [4]. Longer lasting impairment and muscular dysfunction may be a consequence of DOMS [6]. Generally accepted epidemiologic data regarding its incidence and prevalence is missing.

The physiological basis of muscle contraction and damage draws upon the relationship between force, speed and tension in a muscle fibre. Constant velocity lengthening produces a complex tension record, leading to a continued increase in tension throughout a given movement [7]. Beyond the plateau of the length tension curve, stretching the muscle works non-uniformly, elongating the weakest sarcomeres first, which are those with the smallest number of crossbridges [7]. As a consequence, disturbances of the cross-striated band pattern of the sarcomere can be observed. Biopsies taken after repetitive eccentric muscle actions have revealed broadening, streaming, and at times, total disruption of Z-discs [8]. Eccentric exercise led to a dramatic change in the arrangement of the t-tubule network and the disposition of the triads, which is thought to be a primary reason for disorders of the membrane systems involved in excitation-contraction coupling failure [9]. Microscopic disruptions are also thought to lead to other damaged cell membranes and, therefore, a loss of intracellular calcium homeostasis [10]. Several inflammatory processes are initiated, which in turn lead to fiber damage and cell death [5]. Exercise induced muscle damage (EIMD) is variable in severity in muscles with different architecture, and is exacerbated by inflammation after the initial injury [11].

Still, the debate about why this pathophysiologic response mediates pain remains controversial. There is no doubt that excessive exercise, triggered by frequency or load, can be painful [12]. However, there is no consensus regarding the origin of pain. From a clinical point of view, this understanding is crucial in order to choose adequate preventive and rehabilitative concepts.

Whereas some researchers suggest e.g. muscular fascia to cause pain [13], others showed the presence of cutaneous allodynia [14]. Whilst muscular fascia would just need to induce local nociceptors (peripheral sensitivity), it is also conceivable that both observations are the result of an increased spinal sensitivity [15]. In addition, the breakdown products of the impaired muscle tissue excites nociceptors. Specifically, the transient receptor potential channels of the vanilloid type TRPV1 and acid sensing ion channels ASICs have been suggested to play a central role in the processing of the stimuli [16]. In combination with an augmented mechanical response in muscle thin-fibre sensory receptors [17], these neurophysiological observations could be related to muscle tenderness and soreness.

Pain is initiated through the activation of nociceptors by noxious thermal, mechanical and chemical stimuli [18]. There are two classes of nociceptors, myelinated Aδ fibres that mediate acute, well-localized “first” or fast pain, and unmyelinated C fibres mediating poorly localised, “second” or slow pain [19]. Whereas C-fibres develop mechanical sensitivity only in the setting of injury [20], Aδ nociceptors also respond to chemical and thermal as well as mechanical stimuli. A nociceptor response is primarily linked to the occurrence of a tissue injury, and can be considered at least partially as the result of modification of the extracellular milieu by intracellular contents released from injured cells, such as protons, potassium ions or ATP [21]. On the muscular cellular level, almost half of the muscle fibres present low threshold mechanosensitive units (LTM, on Aδ fibres) which respond to weak stimuli such as deformation of the muscle tissue and fascia [22]. They have been implicated as the primary afferents transmitting signals to or maintaining sensitization of wide dynamic range neurons [23] which are likely involved in deep, spread and referral pain. Muscle nociception is supposed to involve high-threshold mechanosensitive receptors (HTM, on C and Aδ-fibres). Their membrane may be equipped with transient receptor potential channels that seem to play pivotal roles in the processing of the stimuli [16, 24, 25]. Other candidates of mechanotransducers include members of the degenerin/epithelial Na+ channel (DEG/ENaC) families, involving above mentioned ASCIs [26]. Hoheisel et al. showed that the activation of these HTM units is pressure dependent and requires a certain pressure threshold to be exceeded [27]. The receptors are supposed to be first hyper-sensitized by chemical mediators like bradykinin and prostaglandin-2, and secondly to be activated by the increased interstitial pressure caused by the development of intramuscular swelling due to DOMS-related oedema [28]. Once activated, the pain signal is transduced by the activation of a variety of voltage-gated ion channels, with the critical role of sodium and potassium channels to convey the signal to the dorsal horn [19]. The signal is mediated to the CNS, place of the sensory, emotional and affective assessment, leading to enhanced processing of nociceptive messages [29]. In persistent pain, it is assumed that the mechanism of peripheral sensitisation results in alteration in the properties of peripheral nerves. This second phase of pain results from inflammation-associated changes in the chemical environment of the nerve fibres [30]. An important role has been contributed to the nerve growth factor NGF, producing hypersensitivity to heat and mechanical stimuli via two temporally distinct mechanisms. First, the sensitivity of the nociceptor is changed, as target proteins functionally potentiate at the peripheral nociceptor terminal; most notably TRPV1, leading to a rapid change in cellular and behavioural heat sensitivity [31]. In addition to this first response, NGF promotes increased expression of pro-nociceptive proteins in the nucleus of the nociceptor (for review [19]). Together, these changes in gene expression enhance excitability of the nociceptor and amplify the neurogenic inflammatory response.

To investigate different mechanical pain thresholds, standardised methods of quantitative mechanical sensory testing have been established [32], allowing to distinguish cutaneous perception (A-beta fibres assessed with thin filaments) from superficial (LTM-evoked, A-delta fibre mediated hyperalgesia to pinprick stimuli) or deep tissue nociception (HTM evoked, C- and A-delta fibre mediated pain to pressure). The present study has been designed to test the hypothesis if the HTM units represent the primarily involved nociceptor in DOMS.

## Methods

### Study design

A single-centre, randomised study at the Sports Campus, Goethe-University Frankfurt to investigate mechanical pain and detection thresholds of the non-dominant biceps brachii muscle following DOMS in healthy adults. Participants were assessed for study eligibility using the following exclusion criteria: DOMS within last 7 days, pregnancy and lactation, severe illness limiting physical or psychological health, and frequent intake of analgesics. Participants signed a written informed consent to participate in the study, which has been approved by the Ethics Committee FB05 of the Goethe University of Frankfurt, Germany (reference 2015–157) and is in agreement with the Declaration of Helsinki (Version Fortaleza 2012). After enrolment, participants were subsequently randomised to either Group 1 or Group 2. Groups differed regarding the sequence of the threshold assessments, i.e.

Group 1: Mechanical Detection Threshold (MDT) → Mechanical Pain Threshold (MPT) → Pressure Pain Threshold
Group 2: Pressure Pain Threshold → Mechanical Detection Threshold (MDT) → Mechanical Pain Threshold (MPT)

The rationale behind allocating the participants into two groups focuses on the possible role of spinal pain inhibiting/enhancing mechanisms. The alternation of the sequence of measures might reveal possible between-measure mechanism.

After the initial baseline measures, DOMS was induced and measures were repeated immediately thereafter and every 24 hours, up to 72 hours post induction. The main outcome measure was the PPT over the biceps muscle belly. Secondary outcome measures were the MDT and MPT, the pain intensity as rated on a visual analogue scale (VAS), the pain perception as assessed with the McGill-short form questionnaire and the maximum isometric voluntary force (MIVF) of the elbow flexors. Participants were told not to exercise during participation in the study and not to use DOMS alleviating treatments.

### Randomisation

Participants were randomly assigned to one of the two study groups using the smartphone-based application Certified True Randomizers (Integer Generator, Random.org, Dublin, Ireland).

### Sample size estimation

Sample size was estimated using the software G*Power (Version 3.15, University of Düsseldorf, Germany). With α set at 5%, 18 participants are required to have 80% power to detect a difference in pressure pain threshold of 11% from baseline between groups [33]. Taken a drop out ratio of 10% into account, twenty participants were estimated to be included into the study.

### Induction of DOMS

At baseline, DOMS of the non-dominant elbow flexors was experimentally induced adhering to a standardised protocol [34]. All participants were seated at a preacher’s bench (multi muscle machine m3, Diagnos^+^,Schnell Trainingsgeräte GmbH, Peutenhausen, Germany), with the hand in supination and with the upper arm, wrist, and shoulder fixed in a way allowing to perform standardised, isolated and controlled biceps curls. Following a standardised warm-up exercise for 3 minutes, i.e. eccentric and concentric movements of the arm without loads, individual one repetition maximum (1 RM; i.e., the maximum weight lifted with one concentric contraction), was determined for the elbow flexors within a maximum of five trials by loading the dumbbell with free weights in 0.5 kg increments. Participants were encouraged verbally to elicit their maximal effort. Ninety percent of the concentric 1 RM was then used to provoke DOMS through eccentric contractions. For this, the dumbbell was lifted by the experimenter, until the participant’s elbow was flexed approximately 120°, and the participant then had to lower the weight eccentrically over a time span of 5 seconds, controlled by a typical metronome, until the elbow was extended (~0–5°). Participants completed a maximum of 6 sets, each comprising 5 repetitions and 2 minutes resting time between sets. The procedure was stopped if the participants could not control lowering the weight in time more than twice in a row in two consecutive sets.

### Outcome measures

Demographics included height (in cm), weight (kg), level of activity (hours), time spent with training (hours), etc.), as well as the previous experience of DOMS on a 4-fold Likert scale (1–2 per year; monthly; weekly; >2 per week).

Mechanical Testing was performed according to the recommendations of the German Research Network on Neuropathic Pain [32], comprising mechanical detection thresholds (MDT), mechanical pain thresholds (MPT), and pressure pain thresholds (PPT). A comprehensive summary of the mechanical measures of the somatosensory system has been described elsewhere [32]. Testing was performed at five equidistant points, perpendicular to the individuals’ belly of the biceps brachii muscle. On a thought line between the tuberositas radii and the coracoid prominence, seven sections were determined by using a tape measure and dividing the belly into seven sections. The beginning of the first (tendon) and the end of the last section (insertion) were not considered being muscle points. Distance between points was according to individual anthropometrical characteristics. Points were marked with a waterproof and skin tolerant pen to ensure the same spots of measurement during the study, as previously reported [33, 35]. Testing always started proximally.

MDT was measured with a set of von Frey filaments, incrementing by a factor of 2 from 0.25 mN to 512 mN (Marstock-nervtest Ltd., Marburg, Germany). The geometric mean of 5 ascending and 5 descending series of stimuli (1-second duration per stimulus) generated the mechanical detection threshold.

MPT was measured with a set of 7 weighted pinprick stimulators each with a blunt contact area of 25 μm diameter (MRC Systems GmbH, Heidelberg, Germany) [36]. The intensity of the punctate stimulators is incremented by a factor of 2 from 8 to 512 mN. The method of limits was used to determine the intensity at which participants distinguished between prick and blunt touch.

The PPT was assessed with a mechanical pressure algometer (pdt, Rome, Italy; range 2–20 kg/cm^2^, diameter 1 cm). The algometer was applied to each of the above described points with increasing force at a rate of approximately 1 kg/cm^2^ per second until the participant reported a painful sensation and the force value was recorded (kg/cm^2^). We explained to each participant to differentiate between tenderness and the feeling of pressure versus real pain. After the muscle was investigated at all five points (from proximal to distant), the subjects were allowed to rest for 5 min. This procedure was repeated a total of 3 times, following the protocol as proposed by Park [37].

Maximum isometric voluntary force (MIVF) of the biceps muscle was measured using the m3 (multi muscle machine; Diagnos^+^, Schnell Trainingsgeräte GmbH, Peutenhausen, Germany) with the elbow flexed at 90° and pressing the wrist against a bar connected to a force transducer (100 Hz sampling rate). Three trials were performed with contractions lasting 5 seconds, separated by 2 minute rest intervals. Participants were encouraged verbally to elicit their maximal effort and force was displayed on a visual display in real time providing immediate feedback. Peak force values (N) were recorded, and the highest of the three repetitions was used for statistical analysis.

Pain intensity at rest and during active movement (flexion and extension) of the biceps muscle was assessed using a visual analogue scale VAS ranging from 0 to 10 cm (with 0 indicating no pain and 10 experiencing the worst imaginable pain).

Participants completed the Short Form McGill Pain Questionnaire (SF-MPQ) for sensations experienced in the non-dominant biceps muscle. The SF-MPQ was adapted to German language and we used ten sensory and four affective pain descriptors that could be ranked in intensity from 0 = none to 3 = severe [38]. The sum of ranked values provides a sensory (SPRI; 0–30), an affective (APRI; 0–12) and a total pain score (TPRI; 0–42).

### Statistics

Statistical analysis was conducted for comparison of the main and secondary outcome measures between the study groups. After testing for normal distribution, the following parametric methods were used: 1) unpaired t-tests for the comparisons between both study groups and 2) repeated measures analysis to analyse effects over time in the overall group. Sphericity of the data was tested with Mauchly’s test and if assumption of sphericity was been violated, Greenhouse-Geisser was applied for correction. Post hoc tests to compare the different points in time were done with paired t-tests. The Bonferroni test was applied to compensate for multiple measurements (in total n = 6), with p < $\frac{0.05}{6}$ = 0.0083 for intragroup analysis. Correlations were calculated according to Pearson for parametric data. Demographic data are presented as mean ± standard deviation and outcome measures as mean ± standard error of the mean. We calculate the value of Cohen's d using the means and standard deviations of two groups to estimate the effect size of the greatest change to baseline.

Data analysis was performed with the SPSS statistical software system, version 23.0 (SPSS Inc., Chicago, IL, USA).

## Results

Twenty participants (9 female and 11 male, age 25.2 ± 3.2 years, weight 70.5 ± 10.8 kg, height 177.4 ± 9.4 cm) were included in the study (for details see Table 1). One dropout occurred at the last visit (72 hours) due to scheduling conflicts.

**Table 1:** Demographics Table 1 summarizes the relevant baseline characteristics of the included subjects. Data is indicated as mean ± SD. No significant baseline differences have been detected between groups.

	All (n = 20)	Group 1 (n = 10)	Group 2 (n = 10)
Gender (n, female /male)	9/11	5/5	4/6
Age (years)	25.2 ± 3.2	24.5 ± 1.1	25.7 ± 4.5
Height (cm)	177.4 ± 9.4	177.8 ± 12.0	177.0 ± 6.6
Weight (kg)	70.5 ± 10.5	71.3 ± 13.0	69.7 ± 7.9
Main sport activity (n) Endurance training Resistance training Team sports Racket sports None	11 3 3 2 1	4 2 2 2 0	7 1 1 0 1
Time spent with main sport (h)	4.7 ± 3.4	4.4 ± 3.6	5.1 ± 3.4
Time spent with resistance training (h)	3.1 ± 3.1	3.1 ± 3.4	3.1 ± 3.1
Experience of DOMS (n) 1–2 per year Monthly Weekly >2 per week	3 8 7 2	1 4 3 2	2 4 4 0

DOMS was induced in both groups with no difference in eccentric load (Group 1: 10.8 ± 6.5 kg; Group 2: 9.3 ± 6.4 kg; p = 0.613). In both groups there was one subject being exhausted after 5 sets, and all others completing the whole protocol. We found significant time effects for all pain related outcome measures as well as for the MIVF, indicating that we had been able to provoke the development of DOMS (see below, and S1 Table).

There were no significant differences as a consequence of the sequence of threshold detection, nor depending on the sites of measure (see S2 Table). Therefore, data was pooled for the following statistics.

### Pressure pain threshold

**Fig 1:**
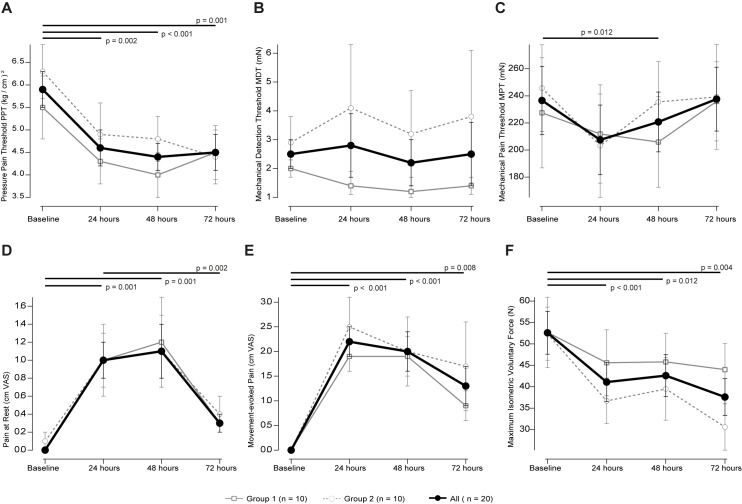
A-C display the mechanical thresholds at baseline and 24, 48 and 72 hours after induction of DOMS. Mean change ± SEM of the PPT in kg/cm^2^ (A), the MDT in mN (B) and the MPT in mN (C). D-F display pain and DOMS-related dysfunction. Mean change ± SEM of pain at rest (A) and during movement (B) in cm VAS and maximum isometric voluntary force (C). Black lines with p-values indicate the post-hoc tests between two times in case that repeated measures ANOVA detected significant effects x time. No between group differences (i.e. sequence of sensory testings) could be observed (refer to the text).

The two-way repeated-measure ANOVA revealed a significant effect for time (F = 175.528 p < 0.001) but no time × sequence of pressure pain threshold assessment (F = 0.667 p = 0.43). Post hoc test revealed significant decrease to baseline at 24 hours (paired t-test p = 0.002), at 48 hours (p < 0.001) and at 72 hours (p = 0.001; Fig 1A). The maximum change in PPT was achieved at 48 hours: -1.5 ± 1.4 kg/cm^2^ (-24%; Cohen’s d = 0.86).

### Mechanical detection threshold

The two-way repeated-measure ANOVA revealed a significant effect for time (F = 7.980 p = 0.012) but no time × sequence of mechanical detection threshold assessment (F = 1.295 p = 0.27). Post hoc test revealed no significant differences to baseline (Fig 1B).

### Mechanical pain threshold

The two-way repeated-measure ANOVA revealed a significant effect for time (F = 110.707 p < 0.001) but no time × sequence of mechanical pain threshold assessment (F = 0.061 p = 0.81). Post hoc test revealed a statistical trend towards an increase to baseline at 48 hours (-15.9 ± 82.9 mN; paired t-test p = 0.012; Cohen’s d = 0.15; Fig 1C).

### Pain intensity

The pain intensity (VAS) during movement (F = 35.268 p = 0.001) and at rest (F = 14.913 p = 0.002) significantly increased over time with no differences between groups. “Pain at rest” was significantly increased comparing baseline to 24 and 48 hours (both p = 0.001) and when comparing 72 to 24 hours (p = 0.002). Differences to baseline at 72 hours (p = 0.018), and when comparing 24 to 48 hours (p = 0.015; Fig 1D) were not significant. “Pain during movement” was significantly different to baseline at 24 and 48 hours (both p < 0.001) and at the limit of the level of significance at 72 hours (p = 0.008; Fig 1E). The maximum pain intensity during movement was 2.2 ± 1.4 cm VAS after 24 hours (Cohen’s d = -2.1) and at rest 1.1 ± 1.2 cm VAS after 48 hours (Cohen’s d = -1.2).

### Mean isometric voluntary force

The two-way repeated-measure ANOVA revealed a significant effect for time (df1 F = 100.006 p < 0.001), but not for time × sequence for the assessment of mean isometric voluntary force (F = 0.701 p = 0.41; Fig 1B). The reduction of MIVF was close to significance at 48 hours (p = 0.012), and significantly reduced at 24 (p < 0.001) and 72 hours (p = 0.004; Fig 1F) when compared to baseline. The maximum decrease in force was -16.8 ± 21.1 N after 72 hours (Cohen’s d = 0.72).

### Correlation between mechanical sensory thresholds and pain intensity or voluntary force

**Fig 2:**
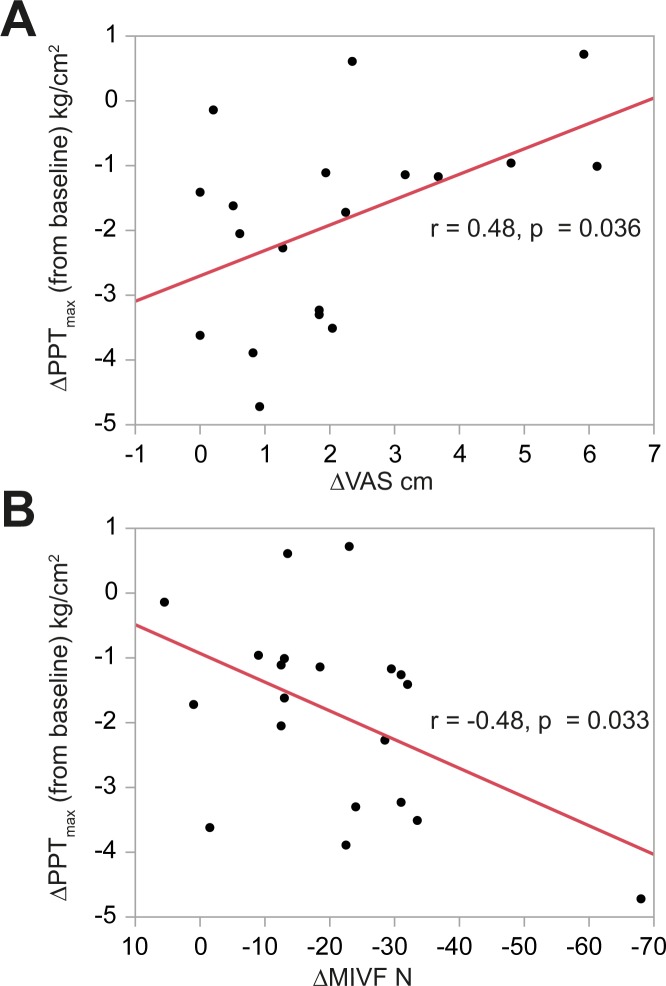
Displays the correlation between the change in PPT (in kg/cm^2^, with negative numbers indicating an increase in pressure pain) and the change in maximum pain intensity (in cm VAS, A) or MIVF (in N, B).

Linking pain to mechanical thresholds indicated a correlation between the maximum change in PPT and the respective change in pain intensity (r = 0.48, p = 0.036, Fig 2A). No correlations between the maximum change in MDT (r = -0.1, p = 0.68) or MPT (r = -0.28, p = 0.23) and the respective change in pain intensity could be detected. Linking pain to function indicated a negative correlation between the maximum change in MIVF and the respective change in PTT (r = -0.48, p = 0.033; Fig 2B), but not MIVF and pain intensity (r = 0.04, p = 0.86).

### Sensory description of DOMS

**Fig 3:**
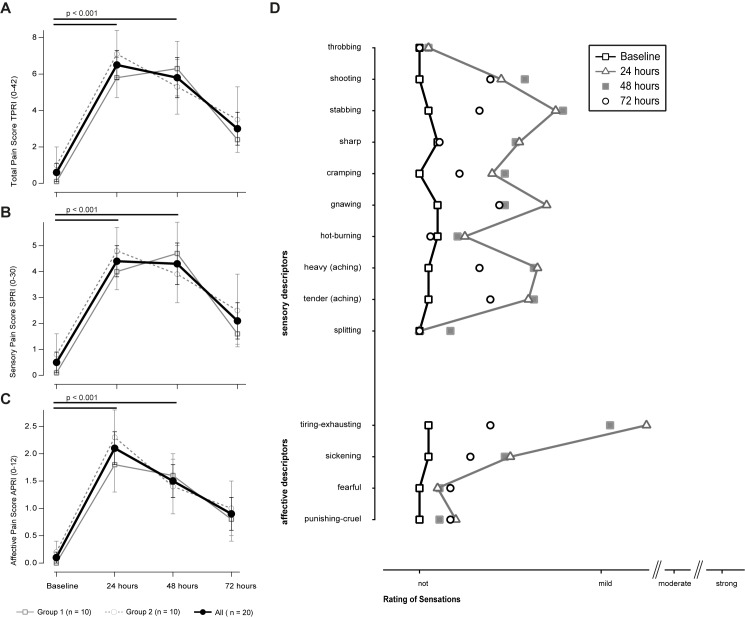
A-D display the rating of pain perception on the short-form MQP. (A)-(C) shows the mean change ± SEM at baseline and 24, 48 and 72 hours after induction of DOMS of the Total (A), Sensory (B) and Affective (C) Pain Score. D lists the used descriptors ranging from not to strong at the 4 time points. Descriptors at baseline and after 24 hours were connected with lines for a better visualization. Black lines with p-values indicate the post-hoc tests between two times in case that repeated measures ANOVA detected significant effects x time. No between group differences (i.e. sequence of sensory testings) could be observed (refer to the text).

The SF-MPQ was used to assess the intensity and quality of sensations arising from the induction of DOMS. Under baseline conditions, the total pain rating index (TPRI) was 0.6 ± 0.5 with a sensory (SPRI) and affective component (APRI) scores of 0.1 ± 0.1 and 1.0 ± 1.0 respectively (Fig 3A). The two-way repeated-measure ANOVA revealed a significant effect for time for the TPRI and both subscores (all p < 0.001), but not for time × sequence (all p > 0.05).

One day after the induction of DOMS the total intensity rating significantly increased to 6.5 ± 3.7 (paired t-test, p < 0.001; Cohen’s d = -1.9), and was significantly increased at 48 (p < 0.001), but not at 72 hours (p = 0.048). The SPRI and the APRI did significantly increase in this regard, Fig 3B and 3C).

The SF-MPQ offers descriptors to qualitatively describe sensations. Under baseline conditions, none of the ratings was reasonably affected. Following DOMS, it were the sensory descriptors stabbing, gnawing, heavy and tender aching the descriptors most likely to be categorised ‘mild painful’. The average rating of ‘throbbing’, ‘shooting’, ‘stabbing’, ‘sharp’ and ‘hot-burning’ during ischemia was mild-moderate. The affective descriptors, ‘tiring-exhausting’ were rated mild, too (Fig 3D).

### Effects of gender

As there were no sequence effects neither for male or female participants, data was consequently pooled. Pain and DOMS-related dysfunction significantly occurred independently of gender (female n = 9). However, two-way repeated-measure ANOVA revealed a significant effect for time x gender of PPT (df1 F = 30.447 p < 0.001) and MIVF (df1 F = 57.480 p < 0.001), with a more pronounced decrease in PPT in female, and of MIVF in male participants. Post-hoc tests did not reveal significant differences at the respective times.

## Discussion

We present the results of a study investigating the effects of DOMS on different sensory and functional outcomes. Our DOMS model was successfully applied as evidenced by the increase in pain threshold, pain intensity and functional impairment. The results are in line with previous investigations, showing a similar increase in pain and loss of function in the subsequent 48 hours following exercise [12, 33, 35, 39, 40]. In contrast to previous studies, we implemented different mechanical pain thresholds, to differentiate the origin of perceived pain. Thus, we were able to demonstrate that the PPT was the only mechanical pain measurement that correlated significantly with the perceived participative muscle pain intensity. The PPT showed the largest effect regarding the quantification of pain. All results together consolidate the hypothesis of an intramuscular pain origin.

In view of our results, the decrease in PPT of approximately 24% supports the idea that muscular HTM receptors play a primary role in the induction of pain. The proportion of HTM receptors is increased in type IV (non myelinated nerve fibres) when compared to type III (myelinated) fibres [41]. A high mechanical threshold (e.g. strong pressure) is required to evoke a receptor potential [27, 42]. In contrast, LTM units respond to any form of innocuous muscle sensations. Several conditions have been shown to increase the mechanical sensitivity of the HTM nociceptors. First, the spread of endogenous substances (bradykinin, nerve growth factor NGF, TNF-α and others) following mechanical tissue damage [41, 43]. This increase is maximised when substances act in concert [44]. Second, a subpopulation of nociceptors can be sensitised and activated during muscle work under ischemic conditions [41]. Third, inflammation causes receptors to become more susceptible to weak stimuli [45]. Strong or long-lasting inflammation is supposed to alter the proportion of HTM receptors towards LTM units, indicating a lowered mechanical threshold in inflamed muscle.

We could demonstrate that a decrease in PPT (i.e. facilitated elicitation of pressure pain) strongly correlates with a reduced perceived pain intensity. This is astounding, as many studies thought PPT and VAS express the same characteristics of pain in DOMS. However, a decrease in PPT strongly correlates with reduced maximum isometric voluntary force. One possible conclusion from these observations is that the typical discomfort as described in DOMS [4, 5] is more likely evoked in low threshold mechanosensitive units as a response to weak stimuli such as deformation of the muscle tissue and fascia [22]. This assumption could not be confirmed from our results, as there is no correlation between the maximum change in MPT and the respective change in pain intensity, indicating that a reduced mechanical pain threshold (addressing such LTMs) does not necessarily increase the pain intensity. The PPT in contrast addresses other mechanism of muscle nociception, i.e. high-threshold mechanosensitive receptors, which is more likely to be linked with muscular dysfunction. From an athlete’s point of view, this differentiation may be crucial. In daily routine, athletes complain more often about muscular dysfunction and interruption of training or competition, than about general discomfort. The differentiation between superficial and deep pain may thus substantially change the therapeutic strategy. DOMS is classically considered a self-limiting condition that usually requires no treatment [39]. Still, the role of inflammation -a major factor sensitising HTMs- has been postulated being responsible for further EIMD [39]. Whereas subjective pain is an indicator of the early 48 hours, the PPT is still present after 72 hours, which is in line with concepts in EIMD prevention, proposing that the timing of DOMS disappearance occurs prior to complete structural and functional recoveries according to the eccentric and stretch-shortening cycle [46]. The sensory and affective descriptors of DOMS; as assessed in this study, also match the proposed concept of pathophysiology. One could hypothesise from the data, that the first 24 hours are dominated by heavy, sharp discomfort (which would correspond to an activation of Aδ-afferents), whereas the upcoming hours carry dull and diffuse pain (i.e. afferent C-fibres; [47]). DOMS has for long been considered a protective mechanism that already disappears too early. Our data suggest that PPT could be the better diagnostic marker to assess the potential risk of severe EIMD.

The stimuli arising from muscle causing pain are normally projected to spinal neurons of ascending nociceptive pathways to sites in the brain stem and thalamus, e.g. the spinothalamic tract [48]. In addition, the activation of a complex neuronal networks in the dorsal horn contributes to the development of secondary hyperalgesia, i.e. lamina I cells which respond to noxious mechanical or thermal stimuli and lamina IV-V cells receiving polysynaptic nociceptive input from muscle receptors as well as other tissues. These multireceptive neurons (wide dynamic range neurons, WDR) combine input from Aδ and C fibres and can respond to both, low-threshold superficial stimuli and high-threshold noxious stimuli [48, 49]. WDR neurons react to noxious stimuli with a graded increase in discharge [50].

Our results suggest that the spinal multireceptive network modifying and spreading the stimuli in the sense of secondary hyperalgesia (see above) are of subsidiary importance. The MPT was reduced over time by approximately 10%. This is a probable correlate of the combined input from low- and high-threshold noxious stimuli to the spinal horn neurons. The lack of remarkable effects on the MDT makes major excitation of other sensory pathways via spinal pathways less probable. The lack of cutaneous allodynia suggests that DOMS is not a strong enough stimulus to elicit ‘central sensitization’, i.e. an increased responsiveness of nociceptive neurons in the central nervous system. The absolute lack of between-group effects, i.e. the sequence of threshold measures implies that pain arising from HTM receptors is primarily projected to brain sites where pain is perceived.

However, other recent research assumes a contribution of C-tactile fibres to cutaneous allodynia in participants with experimentally induced DOMS at the lower leg [14]. Authors suppose that vibration caused cutaneous allodynia as intradermal anaesthesia abolished this effect. This is not in contrast with our data, showing some minor effects on cutaneous Aβ fibres (MDT). This is in accordance with Nagi and Mahns who showed the intensity of allodynia to be 1.7 ± 0.2 cm VAS, which is of minor clinical relevance. At this point it remains unclear whether segmental irritation or anaesthesia may induce mechanism of segmental inhibition to the muscular pain perceived in DOMS. However, a shortcoming of this study is that authors did not investigate the impact of cutaneous anaesthesia on muscular PPT. Thus, mechanical hyperalgesia can be regarded a fortiori the cardinal symptom in DOMS [51].

We are aware that gender differences could potentially influence the findings in sensory studies [52]. In the present investigation, we did not find differences concerning the outcomes indicating the onset of DOMS. We could show that the PPT had a significantly greater decrease in women, as did force in men. Thus, it might be possible that the sensory and functional response to the experimental model might slightly differ. This could potentially influence the primary outcome when PPT or MIVF are chosen. However these observations need to be carefully interpreted due to the small sample size and further research is necessary for verification.

A limitation of our investigation might be that we did not use the full range of the sensory assessment tools as provided by the German Research Network on Neuropathic Pain [32]. However, Queme et al. clearly identified DOMS to influence mechanical but not thermal sensitivity [53]. Thus, in our opinion our methods are appropriate to assess the most relevant mechanical thresholds in DOMS.

The current model applies experiments and measures at the biceps brachii as previous studies could demonstrate its principle role in DOMS. Several factors could influence this model. In regard to the eccentric exercise, the magnitude of contraction-induced EIMD can be influenced by multiple variables, such as fibre length [54, 55], fibre type distribution [56, 57], peak force [58], average force [59], work during the stretch [54], or strain defined as the relative change in length [60–62]. As these factors vary among muscles, assumptions made in this study should be primarily limited to the biceps muscle.

In addition, we are aware that the brachialis muscle is systematically involved with the biceps brachii, and we cannot fully rule out contributing factors in the generation and cure of DOMS. Therefore it could have provided additional valuable data, if e.g. we would have assessed not only the long but also the short head of the biceps muscle. Finally, the role of DOMS-related edema can be a possible contributing factor, however we did not assess intra-muscle pressure or muscle swelling.

In this study we did not assess molecular adaptations on the level of the HTMs and potential inflammatory factors triggering adaptations (e.g. NGF, as well as bradykinin, TNFα, IL-6, or BDNF). To better understand the sensitisation of HTMs, further functional studies should be encouraged to assess these associations. However, these studies will most likely require transgenic animal models to demonstrate cause and effect.

Finally, our study does not answer the question whether treatments addressing the HTMs would be beneficial in the prevention of further damage. Reducing the degree of inflammation and the amount of cytokines irritating the muscular tissue could shorten the period needed to recovery. However, one should also be aware of the potentially delayed structuro-functional recovery when attenuating the inflammatory process [63, 64]. To this point, it could not yet be determined if pain therapy desensitizing the HTM units, i.e. preferentially local deep intramuscular techniques or anti-inflammatory approaches, are beneficial (e.g. enhanced healing) or harmful (e.g. increased risk of EIMD) for the athlete.

## Conclusion

The sensitization of HTM receptors in the muscle seems to be a primary mechanism in the facilitation of DOMS. The PPT is a reliable method to adequately determine the degree of pain. Other spinal or central mechanisms of pain are of subsidiary importance, but could still contribute to the appearance of prolonged states of DOMS. The PPT rather than pain intensity might thus present a potential marker to assess the risk of overloading to early (at a stage of incomplete regeneration) after EIMD.

## Supporting information

S1 Table Outcome measures. Indicates the measures at baseline with no significant differences between groups, and following 24, 48 and 72 hours after the induction of DOMS. Data is indicated as mean ± SD (95%-CI). PPT pressure pain threshold; MDT mechanical detection threshold; MPT mechanical pain threshold; VAS visual analogue scale; MPQ McGill Pain Questionnaire; TPRI: total pain rating index (range 0–42); SPRI sensory pain rating index (0–30); APRI affective pain rating index (0–12); MIVF maximum isometric voluntary force.
*post-hoc tests revealed no significant differences between times.
(DOCX)

S2 Table Measure sites. Testing was performed at five equidistant points, perpendicular to the individuals’ belly of the biceps brachii muscle. On a thought line between the tuberositas radii and the coracoid prominence, seven sections were determined by using a tape measure and dividing the belly into seven sections. The beginning of the first (tendon) and the end of the last section (insertion) were not considered being muscle points. The table indicates the measures at baseline with no significant differences between groups, and following 24, 48 and 72 hours after the induction of DOMS at the five measure sites. No differences in thresholds between sites over time could be detected. Data is indicated as mean ± SD (95%-CI). PPT pressure pain threshold; MDT mechanical detection threshold; MPT mechanical pain threshold.
(DOCX)
