# Supplementary material for: The pain threshold of high-threshold mechanosensitive receptors subsequent to maximal eccentric exercise is a potential marker in the prediction of DOMS associated impairment
Source: PLoS One. 2017 Oct 6;12(10):e0185463. doi: 10.1371/journal.pone.0185463 (PMC5630131; doi:10.1371/journal.pone.0185463)
Supplement: S1 Table — Indicates the measures at baseline with no significant differences between groups, and following 24, 48 and 72 hours after the induction of DOMS. Data is indicated as mean ± SD (95%-CI). PPT pressure pain threshold; MDT mechanical detection threshold; MPT mechanical pain threshold; VAS visual analogue scale; MPQ McGill Pain Questionnaire; TPRI: total pain rating index (range 0–42); SPRI sensory pain rating index (0–30); APRI affective pain rating index (0–12); MIVF maximum isometric voluntary force. *post-hoc tests revealed no significant differences between times. (DOCX) [file pone.0185463.s001.docx]

**S1 Table. Outcome measures.**

|  | **Baseline** | **24 hours** | **48 hours** | **72 hours** | **ANOVA**  repeated-measures |
| --- | --- | --- | --- | --- | --- |
| **PPT (kg/cm^2^)** | 5.9 ± 1.97  (4.98;6.83) | 4.57 ± 1.86  (3.71;5.44) | 4.39 ± 1.52  (3.67;5.1) | 4.48 ± 1.75  (3.63;5.32) | <.001 |
| **MDT (mN)** | 2.47 ± 2.22  (1.43;3.51) | 2.76 ± 5.01  (0.41;5.1) | 2.22 ± 3.52  (0.57;3.86) | 2.5 ± 4.89  (0.15;4.86) | .012* |
| **MPT (mN)** | 236.57 ± 113.01  (183.68;289.46) | 207.5 ± 114.78  (153.78;261.21) | 220.7 ± 98.7  (174.51;266.9) | 237.57 ± 105.51  (188.19;286.95) | <.001* |
| **Pain_at rest_**  **(cm VAS)** | 0.04 ± 0.14  (-0.03;0.11) | 1.03 ± 1.06  (0.53;1.52) | 1.12 ± 1.26  (0.49;1.75) | 0.31 ± 0.48  (0.07;0.55) | .002 |
| **Pain_at move_**  **(cm VAS)** | 0.02 ± 0.09  (-0.02;0.06) | 2.21 ± 1.47  (1.52;2.89) | 1.98 ± 1.83  (1.09;2.86) | 1.3 ± 1.89  (0.39;2.21) | <.001 |
| **MIVF (N)** | 52.55 ± 22.32  (41.69;63.78) | 41.13 ± 20.79  (30.84;51.43) | 42.6 ± 21.83  (31.94;53.54) | 37.63 ± 18.75  (28.59;46.67) | <.001 |
| **MPQ_TPRI_** | 0.55 ± 2.24  (-0.5;1.6) | 6.45 ± 3.75  (4.7;8.2) | 5.8 ± 4.75  (3.58;8.02) | 2.95 ± 4.2  (0.98;4.92) | <.001 |
| **MPQ_SPRI_** | 0.45 ± 1.79  (-0.39;1.29) | 4.4 ± 2.64  (3.16;5.64) | 4.3 ± 3.5  (2.66;5.94) | 2.05 ± 3.12  (0.59;3.51) | <.001 |
| **MPQ_APRI_** | 0.1 ± 0.45  (-0.11;0.31) | 2.05 ± 1.5  (1.35;2.75) | 1.5 ± 1.4  (0.85;2.15) | 0.9 ± 1.29  (0.29;1.51) | <.001 |
