# Supplementary material for: The pain threshold of high-threshold mechanosensitive receptors subsequent to maximal eccentric exercise is a potential marker in the prediction of DOMS associated impairment
Source: PLoS One. 2017 Oct 6;12(10):e0185463. doi: 10.1371/journal.pone.0185463 (PMC5630131; doi:10.1371/journal.pone.0185463)
Supplement: S2 Table — Testing was performed at five equidistant points, perpendicular to the individuals’ belly of the biceps brachii muscle. On a thought line between the tuberositas radii and the coracoid prominence, seven sections were determined by using a tape measure and dividing the belly into seven sections. The beginning of the first (tendon) and the end of the last section (insertion) were not considered being muscle points. The table indicates the measures at baseline with no significant differences between groups, and following 24, 48 and 72 hours after the induction of DOMS at the five measure sites. No differences in thresholds between sites over time could be detected. Data is indicated as mean ± SD (95%-CI). PPT pressure pain threshold; MDT mechanical detection threshold; MPT mechanical pain threshold. (DOCX) [file pone.0185463.s002.docx]

**S2 Table. Measure sites**

|  | Measure site 1 | Measure site 2 | Measure site 3 | Measure site 4 | Measure site 5 | **ANOVA**  site x time |
| --- | --- | --- | --- | --- | --- | --- |
| **PPT (kg/cm^2^)** |  |  |  |  |  |  |
| Baseline | 5.47 ± 2.13  (4.48;6.47) | 6.11 ± 1.93  (5.21;7.02) | 5.55 ± 2.16  (4.53;6.56) | 5.85 ± 1.96  (4.93;6.76) | 6.53 ± 2.51  (5.36;7.71) | F = 1.350  p = .251 |
| 24 hours | 4.78 ± 2.12  (3.79;5.77) | 4.92 ± 1.85  (4.05;5.78) | 4.09 ± 1.49  (3.39;4.78) | 4.31 ± 2.37  (3.21;5.42) | 4.78 ± 2.47  (3.62;5.93) |  |
| 48 hours | 4.09 ± 1.66  (3.31;4.86) | 4.51 ± 1.88  (3.63;5.39) | 3.94 ± 1.64  (3.17;4.71) | 4.58 ± 1.94  (3.67;5.48) | 4.83 ± 1.73  (4.02;5.64) |  |
| 72 hours | 4.07 ± 1.67  (3.26;4.88) | 4.58 ± 1.9  (3.66;5.5) | 4.08 ± 1.82  (3.2;4.95) | 4.7 ± 1.97  (3.74;5.65) | 4.96 ± 2.01  (3.99;5.93) |  |
| **MDT (mN)** |  |  |  |  |  |  |
| Baseline | 3.3 ± 2.58  (2.09;4.51) | 2.13 ± 2.3  (1.06;3.21) | 3.99 ± 4.78  (1.75;6.23) | 1.43 ± 1.62  (0.68;2.19) | 1.48 ± 1.1  (0.97;2) | F = 1.414  p = .227 |
| 24 hours | 3.46 ± 4.92  (1.15;5.76) | 2.52 ± 4.95  (0.2;4.84) | 2.52 ± 4.85  (0.25;4.78) | 2.7 ± 4.93  (0.4;5.01) | 2.58 ± 5.79  (-0.13;5.29) |  |
| 48 hours | 2.52 ± 3.07  (1.09;3.96) | 2.32 ± 4.86  (0.05;4.59) | 2.08 ± 3.89  (0.26;3.9) | 2.17 ± 4.89  (-0.12;4.46) | 1.64 ± 2.06  (0.67;2.6) |  |
| 72 hours | 2.09 ± 2.65  (0.82;3.37) | 2.45 ± 4.97  (0.05;4.84) | 2.78 ± 5 (0.37;5.19) | 2.75 ± 6.56  (-0.41;5.91) | 2.45 ± 5.48  (-0.19;5.09) |  |
| **MPT (mN)** |  |  |  |  |  |  |
| Baseline | 249.39 ± 123.11  (191.78;307.01) | 257.45 ± 120.62  (201;313.9) | 220.82 ± 139.9  (155.35;286.3) | 246.42 ± 139.89  (180.95;311.89) | 208.77 ± 142.12  (142.26;275.28) | F = .859  p = .512 |
| 24 hours | 266.3 ± 119.27  (210.47;322.12) | 201.04 ± 146.81  (132.33;269.75) | 192.01 ± 142.37  (125.37;258.64) | 203.7 ± 135.31  (140.37;267.03) | 174.44 ± 129.17  (113.98;234.89) |  |
| 48 hours | 267.35 ± 91.1  (224.71;309.98) | 208.4 ± 127.93  (148.53;268.28) | 214.64 ± 125.11  (156.09;273.2) | 203.93 ± 129.86  (143.16;264.71) | 209.2 ± 118.26  (153.85;264.55) |  |
| 72 hours | 275.21 ± 105.16  (224.52;325.9) | 253.16 ± 128.01  (191.46;314.86) | 250.88 ± 108.45  (198.61;303.15) | 229.26 ± 122.59  (170.17;288.34) | 241.88 ± 120.33  (183.88;299.88) |  |
